# Availability, variety and distribution of healthy and unhealthy foods and beverages sold at street food stands in Mexico City

**DOI:** 10.1017/S136898002100330X

**Published:** 2021-12

**Authors:** Jose B Rosales Chavez, Meg Bruening, Michael F Royer, Punam Ohri-Vachaspati, Rebecca E Lee, Megan Jehn

**Affiliations:** 1 School of Geographical Sciences and Urban Planning, Arizona State University, 975 S. Myrtle Ave, Coor Hall 5th Floor, Tempe, AZ 85281, USA; 2 College of Health Solutions, Arizona State University, Phoenix, AZ, USA; 3 Center for Health Promotion and Disease Prevention, Edson College of Nursing & Health Innovation, Arizona State University, Phoenix, AZ, USA; 4 School of Human Evolution and Social Change, Arizona State University, Tempe, AZ, USA

**Keywords:** Food environment, Street food, Food availability, Mexico

## Abstract

**Objective:**

To examine differences in the availability, variety and distribution of foods and beverages sold at street food stands (SFS) across neighbourhood income levels in Mexico City.

**Design:**

Cross-sectional.

**Setting:**

Twenty neighbourhoods representing low-, middle- and high-income levels in Mexico City.

**Participants:**

Direct observations of SFS (*n* 391).

**Results:**

The availability of healthy foods such as fruits/vegetables was high in middle- and high-income neighbourhoods, whereas the availability of unhealthy foods such as processed snacks was higher in low-income neighbourhoods. However, statistically significant differences in food availability across neighbourhoods were only observed for dairy and processed snack items (*P* < 0·05). Similarly, differences in variety were only observed for cereal and processed snacks (*P* < 0·05). No statistically significant differences were seen for variety of fruits/vegetable across neighbourhood income levels (*P* > 0·05). No statistically significant differences across neighbourhood income levels were observed for beverage availability and variety (*P* > 0·05). Although street foods and beverages were often distributed near homes, public transportation centres and worksites, no differences were observed across neighbourhood income levels (*P* > 0·05).

**Conclusions:**

Findings suggest that SFS can be a source of both unhealthy foods and healthy foods for communities across neighbourhoods in Mexico City. Additional studies are needed to assess the relationship between street food and beverage availability, and consumption.

Mexico has one of the highest rates of overweight and obesity in the world. Currently, 70·6 % of adult Mexican women and 69·4 % of adult Mexican men are either overweight or obese^(1)^. This high prevalence is a serious public health concern as individuals with overweight and obesity are more likely to develop conditions such as diabetes and CVD^(2,3)^. The types of food venues present in a community largely influence people’s access to and consumption of foods, the quality of people’s diets and their health^(4–10)^. Notably, consuming food items such as fruits and vegetables can prevent negative health outcomes including obesity, CVD and some forms of cancer^(11–13)^. These type of food items are more likely to be found in supermarkets and grocery stores^(14–16)^ and less likely to be found in fast-food restaurants and convenience stores. In comparison, fast-food restaurants and convenience stores have a high availability of highly processed, unhealthy food and beverage items^(7,17–19)^. However, in the Mexican food environment, community members have access to an array of culturally relevant food venues that remain understudied. One of these food venues is street food stands (SFS).

Street foods and SFS are an essential element of food environments and vital sources of food and employment for millions of families in low- and middle-income countries, including Mexico^(20–24)^. The United Nations Food and Agriculture Organization defines street foods as ready-to-eat foods and beverages that vendors sell on the streets^(25)^. Street-food vendors use a variety of media to cook, display, store and transport food items: highly mobile stands such as bicycles and wheelbarrows; semi-mobile stands consisting of portable tables, chairs and cooking ware; and stationary stands, which may stay in one place overnight but can be easily moved to a different location^(22,25,26)^. While most street foods are cooked on-site, some vendors prepare the food at home and transport it to key locations to sell it. Street food is popular due to its affordability and convenience^(20,22,27–30)^, and individuals from all socio-economic backgrounds consume foods and beverages from SFS^(23,27,28,31,32)^.

Even though SFS are an important food source, fewer studies have systematically documented SFS food and beverage availability and variety. In a recent review of 441 SFS studies from around the world, researchers reported that 85 % of the studies focused on food safety, and only 31 % (including several studies that also addressed food safety) discussed food availability^(33)^. Only seven studies addressed aspects of SFS or the availability of street foods and beverages in Mexico^(20,34–39)^. Nonetheless, findings from previous studies in this area have shown that SFS can be a source of healthy items such as fruits, vegetables and water but can also be a source of unhealthy ones, such as processed snacks, regular sodas and other sugar-sweetened beverages (SSB) (e.g. energy drinks, sports drinks, yogurt).

Several limitations have been noted in previous research: (1) using indirect or intermediate methods such as 24-h dietary intake recall to measure usual dietary intake rather than using direct observations^(36,37)^; (2) not using validated assessment tools to objectively document food availability^(20,34,35)^; (3) employing an overly narrow scope in terms of points of access (i.e. place where vendors may be catering) which limits findings to the observed points of access (e.g. schools)^(34,38)^; and (4) not reporting how they chose their SFS sample, which would allow for determination of whether a sample was representative of the broader SFS population, such that a study’s findings could be generalised to this population^(20,39)^. Moreover, none of the studies explored differences in food and beverage availability (i.e. the physical presence of a food/beverage item in a stand), variety (i.e. available varieties or types of the general ready-to-eat food or beverage item per SFS) and distribution (i.e. the physical presence of a food or beverage item within 100 m of a point of access) using validated observational assessment methods. Not using validated objective assessment tools to capture food and beverage availability can result in misleading or biased results that under or over report food availability. Thus, a better understanding of the types of food and beverage items sold at SFS and the availability, variety and distribution of these items across income levels is needed.

Another notable gap in the street food literature is in the area of street food and beverage availability, variety and distribution across income levels and points of access. Studies assessing other types of food venues have shown that food availability varies with neighbourhood income levels and that venues such as fast-food restaurants and convenience stores seem to target low-income, ethnic families^(19,40,41)^. Conversely, supermarkets and grocery stores are more likely to be found in high-income neighbourhoods^(42–44)^. In Asian and African countries, SFS have been associated primarily with low-income communities^(20,32)^, while several other studies have shown that customers from all backgrounds consume street food^(23,27,28,31,32)^. Meanwhile, whether street food vendors in Mexico target customers from specific socio-economic backgrounds is not well documented.

The distribution of food venues and food availability may vary according to points of access (e.g. schools, worksites and other locations with high concentrations of target customers). For example, some studies have found a higher prevalence of fast-food restaurants and convenience stores near schools^(18,40,45,46)^. Unfortunately, children’s access to these food venues has been linked to higher exposure to unhealthy foods and a greater risk for obesity^(40,45,46)^. Two Mexican SFS studies addressed the subject of points of access, but these were limited to schools^(34,38)^. Meanwhile, the relationship between food availability and variety and other points of access (e.g. worksites and transportation centres) or target populations (e.g. low-income neighbourhoods) has not yet been addressed.

Against this backdrop, the objectives of this study were as follows: (1) to document the types of foods and beverages sold at SFS; (2) to describe differences in food and beverage availability and variety across low-, middle- and high-income neighbourhoods; and (3) to describe differences in the distribution of food and beverage items across neighbourhood income levels and points of access located within 100 m of a SFS. Our hypotheses were as follows: (1) there would be a statistically significantly higher availability of healthier food and beverage items (e.g. fruits, vegetables, water) in high-income neighbourhoods and near points of access such as worksites and transportation centres, which tend to have higher concentrations of adults; and (2) there would be a statistically significantly higher availability of unhealthy items (e.g. processed snack items, regular sodas) in low-income neighbourhoods and near points of access such as parks and schools, which tend to have higher concentrations of children. By documenting the types of ready-to-eat food and beverage items sold at SFS, our study explores whether SFS are a source of healthy or unhealthy food items in a Mexican city. Documenting the types of food and beverages sold at SFS is the first step towards understanding the impact of street foods on health outcomes and food security.

## Methods

### Selection of street food stands

A sample of SFS in Mexico City was selected for assessment. In Mexico City, we expected to find a diverse array of ready-to-eat foods and beverages at SFS, representing the cuisine of various regions in Mexico. Given that many street food vendors do not register their business with the local government, it was not possible to use a business directory to draw a random sample of SFS. Therefore, we devised an innovative approach to identify SFS throughout the city, which involved recruiting SFS on particular street segments. To identify street segments for the assessment, we selected a random sample of census tracts representing five different marginalisation levels throughout Mexico City: very high marginalisation (i.e. very low-income), high (i.e. low-income), middle (i.e. middle-income), low (i.e. high-income) and very low (i.e. very high-income). The Mexican government defines marginalisation levels using three domains: education, living arrangements and income. Education is based on the proportion of people 15 years and older in an area who cannot read and by the percentage of people 15 years and older who did not finish elementary school in each locality^(47)^. The living arrangement domain is a composite of the number of households in an area with dirt floors and that lack running water, sewer systems and electricity and by the average number of people per room. Income is defined by the number of individuals in an area who are employed in formal business^(47)^.

We selected a random sample of five census tracts per marginalisation level; henceforth, we refer to marginalisation levels as income levels in this paper. Once the census tracts were randomly selected, we used geographic information system methods to draw a circle (i.e. a buffer) with a 400 m radius around the centre point of each tract. Previous literature has suggested that a buffer of this size is adequate to represent the food environment in a neighbourhood, given that residents are willing to walk for approximately 5 min to reach a food source^(48,49)^. A buffer of this size can also capture various elements of the built environment, including homes, schools, transportation centres, worksites and other locations used as points of access for SFS target populations. Next, we mapped the street segments within each census tract, selecting all arterial street segments for observation but only 25 % of residential ones, as previous research has suggested that residential street segments tend to be quite homogenous. Thus, using 25 % of the residential streets in a census tract can suffice to capture the street segments’ overall characteristics^(50–52)^.

Data collection proceeded as follows. If a stand was not busy with customers, research assistants (RA) would approach the vendor(s) to explain the study’s objectives and to request permission to document the types of ready-to-eat foods and beverages sold at the stand. SFS were excluded from assessments under the following conditions: (1) vendors were selling raw foods meant to be prepared at home (e.g. raw meat stands; fruit/vegetable stands that did not have ready-to-eat items); (2) stands had four permanent walls (e.g. kiosks); and (3) stands were an extension of a store, *fonda* (i.e. mom-and-pop restaurant), or other restaurants. There were no risks associated with participating in the study. However, some vendors seemed uncomfortable upon being approached and declined to participate in the study (*n* 81); they seemed to suspect the RA of being government officials who had come to verify a city permit or conduct a health inspection. Thus, distrust was the main reason that some vendors refused participation. The RA collected most of the information through direct observation, with minimal contact with vendors. They gave vendors a small monetary incentive to encourage their participation but did not collect any vendor personal information. In this observational study, data were collected from May to August of 2018.

### Measures

The data pertaining to the measures in this study were collected using the Street Food Stand Assessment Tool, which was previously validated as capturing SFS characteristics, street food vendors’ points of access and ready-to-eat food and beverage availability and variety (double blind process *et al.*, under review).

### Street food stands characteristics

Selected street segments were randomly assigned to one of the three assessment times: morning, afternoon or evening. Teams of RA walked the full lengths of the street segments moving first north to south and then south to north, east to west and west to east, respectively, within each neighbourhood, searching for SFS. Upon encountering a SFS, an RA would assign a unique identifier to the stand and mark its location on a paper map. Next, the RA would approach the stand and document its basic characteristics, including the stand’s level of mobility (i.e. mobile, semi-mobile or stationary); the stand’s type (i.e. cooked meals, fruits/vegetables, snacks or ‘other’); the vendor’s gender; the neighbourhood’s income level; the street segment’s type; and whether the SFS was a stand-alone business or part of a street market.

### Neighbourhood income

The five income levels were merged into three: the very high- and high-marginalisation levels into the low neighbourhood income level; the very low- and low-marginalisation levels into the high-income neighbourhood income level, while the middle neighbourhood income level remained the same.

### Food availability and variety assessment

The Street Food Stand Assessment Tool is an indicator tool that can be used to record the availability and variety of ready-to-eat foods and beverages sold at SFS, in five food categories (fruit and vegetables, meat, dairy, cereals/grains and snacks/candies) and five beverage categories (regular soda, diet soda, water, natural juice and SSB including flavoured water, coffee, processed juice, energy/sports beverages and dairy beverages). The RA treated availability as a binary variable, recording ‘yes’ when a ready-to-eat food or beverage item was present and ‘no’ when it was not. Variety was recorded as a continuous variable, defined as the available varieties or types of the general ready-to-eat food item. For example, if a stand sold both red and green apples, variety was documented as ‘2’ in the fruit variety category, regardless of the total number of apples at the stand.

### Distribution assessment

Distribution was measured as the physical presence of ready-to-eat food or beverage items within 100 m of a point of access and was recorded as a ‘yes’ or ‘no’ answer. The Street Food Stand Assessment Tool contains a list of different types of venues that can serve as points of access for populations selected by street food vendors. These fall into the following categories: (a) homes, referring to places where individuals or families reside; (b) sports centres, such as gyms and athletic fields; (c) public transportation centres, where people can access buses, subways or trains; (d) *fondas*, referring to small family-owned restaurants (i.e. mom-and-pop restaurants); (e) schools or places where people receive a formal education, such as elementary, middle, high school and college; (f) churches or places of worship; (g) worksites or places of employment; (h) recreational areas or open spaces where children can play (i.e. playgrounds); (i) malls or shopping centres; and (j) restaurants, referring to franchise fast-food restaurants and large sit-in restaurants. The RA recorded all the various points of access within 100 m of the SFS; in other words, an SFS could be located near multiple points of access or venues. For example, a cooked meal stand could be located near a home and a public transportation centre as well as multiple worksites.

### Statistical analysis

The frequencies summarised food and beverage availability, SFS mobility levels, SFS types, vendor gender and points of access within 100 m of the SFS. Chi-square tests of independence were performed to detect differences in ready-to-eat food and beverage availability across neighbourhoods and differences in availability by points of access within 100 m of SFS across neighbourhoods. ANOVA were performed to assess differences in the means of ready-to-eat food and beverage varieties across the three neighbourhood income levels and points of access. An *α* level of 0·05 was used for all statistical tests, and Bonferroni adjustments were performed to account for multiple comparisons. Statistical analyses were performed using Stata statistical software^(53)^.

## Results

### Street food stands characteristics

The SFS characteristics by neighbourhood income level are presented in Table 1. The research team assessed 391 (82·7 %) of 473 identified SFS, with some vendors not giving consent for the RA to document food and beverage availability and variety at their stand (*n* 82). Almost half of vendors selling street food were men (49·4 %). The SFS were more likely to be on residential street segments (51·3 %) than on arterial segments (48·7 %). The highest percentage of SFS assessments (44·6 %) took place during the afternoon observation time, followed by the morning (39·6 %) and evening (15·8 %) observation times. Most SFS were semi-mobile (54·0 %). The most common types of SFS were cooked meal stands (38·1 %), followed by snacks (29·8 %) and fruits/vegetables (19·7 %). The numbers of SFS varied across income levels. Middle-income neighbourhoods had the highest number of SFS (41·9 %), followed by high-income (31·2 %) and low-income (26·9 %) neighbourhoods.

**Table 1:** Street food stands (SFS) characteristics (*n* 391)

SFS characteristics (*n* 391)	Neighbourhood income level							
	Low (*n* 105)		Middle (*n* 164)		High (*n* 122)		Number of SFS	
	*n*	%	*n*	%	*n*	%	*n*	%
Segment publicly accessible								
Yes	105	78·4	164	89·6	122	78·2	391	82·7
No	29	21·6	19	10·4	34	21·8	82	17·3
Type of street segment								
Residential	70	68·0	86	52·4	43	35·5	199	51·3
Arterial	33	32·0	78	47·6	78	64·5	189	48·7
(missing)	2	1·90			1	1·00	3	0·01
Observation time								
Morning	48	45·7	56	34·6	49	41·2	153	39·6
Afternoon	48	45·7	82	50·6	42	35·3	172	44·6
Evening	9	8·57	24	14·8	28	23·5	61	15·8
(missing)			2	1·22	3	2·46	5	0·01
Street food stand categories								
Cooked meals	32	30·5	68	41·5	49	40·2	149	38·1
Snacks	40	38·1	41	25·0	35	28·7	116	29·8
Fruits/vegetables	24	22·9	29	17·7	24	19·7	77	19·7
Other	9	8·57	26	15·8	14	11·5	49	12·5
SFS mobility								
Mobile	34	32·4	56	34·1	27	22·1	117	29·9
Semi-mobile	63	60·0	85	51·8	63	51·6	211	54·0
Stationary	8	7·62	23	14·0	32	26·2	63	16·1
Vendor present at SFS								
Male	49	46·7	84	51·2	60	49·2	193	49·4
Female	44	41·9	60	36·6	46	37·7	150	38·4
Both	12	11·4	20	12·2	16	13·1	48	12·3
Street segments not assessed (*n* 81)								
Observation time								
Morning	21	72·4	13	72·2	17	50·0	51	63·0
Afternoon	5	17·2	5	27·8	10	29·4	20	24·7
Evening	3	10·3	0	0·00	7	20·6	10	12·3

The percentage distribution is based on the observed (non-missing) values, and the percentage missing is based on the total number of observations.

### Street food and beverage availability across neighbourhood income levels

The differences in ready-to-eat food and beverage availability across neighbourhood income levels are shown in Table 2. Statistically significant differences in food item availability across neighbourhood income levels were only observed for dairy (*X*^2^ (2, *n* 391) = 7·68, *P* < 0·05) and processed snack items (*X*^2^ (2, *n* 391) = 8·44, *P* < 0·05). While there were no statistically significant differences for the other items across neighbourhood income levels, SFS in middle-income neighbourhoods had more availability of fruits/vegetables, meat, dairy products and cereals than low- and high-income neighbourhoods. In comparison, low-income neighbourhoods had more availability of unhealthy food items such as processed snacks (34·3 %) than middle- (32·8 %) and high-income (32·8 %) neighbourhoods (*P* < 0·05).

**Table 2:** Differences in food and beverage availability at SFS (*n* 391) across neighbourhood income levels

Type of food or beverage	Neighbourhood income levels						*X*^2^	df	*P*
	Low (*n* 105)		Middle (*n* 164)		High (*n* 122)				
	*n*	%	*n*	%	*n*	%			
Fruits/vegetables (*n* 233)	54	23·2	105	45·0	74	31·8	4·3	2	0·11
Meat (*n* 141)	30	21·3	65	46·1	46	32·6	3·6	2	0·16
Dairy (*n* 102)	18	17·6	53	52·0	31	30·4	7·68	2	0·02*
Cereal (*n* 126)	30	23·8	53	42·1	43	34·1	1·15	2	0·56
Processed snacks (*n* 134)	46	34·3	44	32·8	44	32·8	8·44	2	0·01†
Regular soda (*n* 107)	21	19·6	47	43·9	39	36·4	4·3	2	0·11
Diet soda (*n* 8)	2	25·0	2	25·0	4	50·0	1·49	2	0·47
Water (*n* 34)	6	17·6	13	38·2	15	44·1	3·28	2	0·19
SSB (*n* 90)	21	23·3	35	38·9	34	37·8	2·42	2	0·29

SSB, sugar-sweetened beverages; SFS, street food stands.

* Higher availability in middle- than in low-income neighbourhoods.

† Higher availability in low- than in middle-income neighbourhoods.

Although there were no statistically significant differences in beverage availability across neighbourhood income levels, the following patterns were observed for the different types of beverages. The availability of water was high in high-income (44·1 %) compared to low- (17·6 %) and middle-income (38·2 %) neighbourhoods. The availability of unhealthy beverages such as regular soda was high in middle-income (43·9 %) compared to low- (19·6 %) and high-income (36·4 %) neighbourhoods. A similar observation was made for SSB, with high availability of these beverages in middle-income (38·9 %) compared to low- (23·3 %) and high-income (37·8 %) neighbourhoods. The availability of a less unhealthy soda option such as diet soda was high in high-income (50·0 %) compared to low- (25·0 %) and middle-income (25·0 %) neighbourhoods.

### Street food and beverage variety across income levels

The ready-to-eat food and beverage variety and differences across neighbourhood income levels are presented in Table 3. Processed snack variety differed significantly across income levels: in low-income neighbourhoods, SFS had a higher variety of processed snacks (M = 10·8, sd = 1·84) compared to middle- (M = 4·91, sd = 1·47) and high-income neighbourhoods (M= 9·05, sd = 1·70; *F*_2388_ = 3·55, *P* < 0·05). While there were no statistically significant differences for fruit/vegetable varieties, fruit/vegetable variety was high in high-income neighbourhoods (M = 5·25, sd = 0·47) compared to both low- (M = 4·61, sd = 0·51) and middle-income (M = 5·01, sd = 0·41, *P* > 0·05) neighbourhoods.

**Table 3:** Differences in food and beverage variety in SFS (*n* 391) neighbourhood levels in Mexico City

Food and beverage items	Neighbourhood income levels						ANOVA
	Low (*n* 105)		Middle (*n* 164)		High (*n* 122)		
	Mean	sd	Mean	sd	Mean	sd	*P*
Fruits/vegetables	4·61	0·51	5·01	0·41	5·25	0·47	0·65
Meat	1·28	0·28	2·04	0·23	1·91	0·26	0·10
Cereal	1·08	0·23	0·35	0·19	1·06	0·22	0·02*,†
Dairy	0·53	0·12	0·80	0·10	0·70	0·11	0·23
Processed snacks	10·8	1·84	4·91	1·47	9·05	1·70	0·03*
SSB	1·44	0·30	0·66	0·66	1·61	0·28	0·30
Regular soda	1·07	0·26	1·29	0·20	1·71	0·24	0·16
Water	0·18	0·09	0·24	0·07	0·33	0·08	0·46
Diet soda	0·04	0·03	0·04	0·02	0·08	0·03	0·48

SSB, sugar-sweetened beverages; SFS, street food stands.

* Higher variety in low- than in middle-income neighbourhoods.

† Higher variety in high- than in middle-income neighbourhoods.

Similar to beverage availability, the research team did not observe any statistically significant differences in beverage variety. Some patterns were observed for the different types of beverages. For example, water variety (e.g. brand variety) was high in high-income neighbourhoods (M = 0·33, sd = 0·08) compared to low- (M = 0·18, sd = 0·09) and middle-income neighbourhoods (M = 0·24, sd = 0·07, *P* > 0·05). The regular soda variety was also high in high-income neighbourhoods (M = 1·71, sd = 0·24) compared to low- (M = 1·07, sd = 0·26) and middle-income neighbourhoods (M = 1·29, sd = 0·20, *P* > 0·05). These finding suggests that customers in high-income neighbourhoods may have more beverage options than those in low- and middle-income neighbourhoods.

### Street food and beverage distribution near points of access

The distribution of ready-to-eat foods and beverages varied across points of access and income levels, but these differences were rarely statistically significant. Among all points of access, homes, transportation centres and worksites were the three venues that were consistently reported (at >10 %) within 100 m of SFS (Table 4). The distribution of dairy items near transportation centres was higher in middle-income (56·9 %) than in low-income (20·0 %) neighbourhoods (*X*^2^ (2, *n* 65) = 7·37, *P* < 0·05). The distribution of cereal items was higher near worksites in high-income (61·4 %) than in both low- (15·9 %) and middle-income (22·7 %) neighbourhoods (*X*^2^ (2, *n* 44) = 12·3, *P* < 0·05). SFS selling ready-to-eat fruits/vegetables were often found near homes (47·6 %) and transportation centres (48·2 %) in middle-income neighbourhoods and near schools (50·0 %) and parks (44·1 %) in low-income neighbourhoods, but these differences were not statistically significant (*P* > 0·05). In comparison, SFS selling processed snacks were often found near homes (36·2 %) and transportation centres (37·2 %) in middle-income neighbourhoods and near schools (54·3 %) and parks (43·5 %) in low-income neighbourhoods, but these differences were not statistically significant (*P* > 0·05).

**Table 4:** Distribution of street food found at SFS (*n* 391) across neighbourhood income levels and points of access in Mexico City

Type of food item	Point of access	Neighbourhood income levels						*X*^2^	df	*P*
		Low		Middle		High				
		*n*	%	*n*	%	*n*	%			
Fruits and vegetables										
Homes (*n* 210)		45	21·4	100	47·6	65	31·0	3·92	2	0·14
Sports facilities (*n* 26)		2	7·69	11	42·3	13	50·0	4·14	2	0·12
Transportation centres (*n* 139)		38	27·3	67	48·2	34	24·5	3·30	2	0·19
Food inns (*n* 69)		19	27·5	28	40·6	22	31·9	1·99	2	0·36
Schools (*n* 52)		26	50·0	11	21·1	15	28·8	4·12	2	0·12
Churches (*n* 40)		12	30·0	19	47·5	9	22·5	0·04	2	0·97
Worksites (*n* 82)		13	15·8	33	40·2	36	43·9	1·62	2	0·44
Parks (*n* 34)		15	44·1	10	29·4	9	26·5	1·37	2	0·50
Malls (*n* 24)		0	0·00	22	91·7	2	8·33	–		–
Restaurants (*n* 31)		0	0·00	13	41·9	18	58·1	–		–
Snacks										
Homes (*n* 166)		35	30·2	42	36·2	39	33·6	4·81	2	0·09
Sports facilities (*n* 10)		3	30·0	2	20·0	5	50·0	4·23	2	0·12
Transportation centres (*n* 78)		29	37·2	29	37·2	20	25·6	2·53	2	0·28
Food inns (*n* 34)		12	35·3	15	44·1	7	20·6	1·14	2	0·56
Schools (*n* 35)		19	54·3	8	22·9	8	22·7	0·15	2	0·92
Churches (*n* 28)		10	35·7	14	50·0	4	14·3	1·85	2	0·39
Worksites (*n* 43)		10	23·4	16	37·2	17	39·5	0·82	2	0·66
Parks (*n* 23)		10	43·5	5	21·7	8	34·8	1·92	2	0·38
Malls (*n* 12)		0	0·00	10	83·3	2	16·7	–		–
Restaurants (*n* 21)		0	0·00	8	38·1	13	61·9	–		–
Meat										
Homes (*n* 130)		26	20·0	61	46·9	43	33·1	2·67	2	0·26
Sports facilities (*n* 20)		1	5·00	9	45·0	10	50·0	3·48	2	0·17
Transportation centres (*n* 83)		20	24·1	40	48·2	23	27·7	3·02	2	0·22
Food inns (*n* 40)		12	30·0	11	27·5	17	42·5	8·44	2	0·01‡
Schools (*n* 27)		14	51·8	5	18·5	8	29·6	1·83	2	0·39
Churches (*n* 19)		7	36·8	9	47·4	3	15·8	0·81	2	0·66
Worksites (*n* 51)		8	15·7	18	35·3	25	49·0	2·32	2	0·31
Parks (*n* 22)		9	40·9	6	27·3	7	31·8	0·46	2	0·79
Malls (*n* 13)		0	0·00	12	92·3	1	7·7	–		–
Restaurants (*n* 16)		0	0·00	6	37·5	10	62·5	–		–
Dairy										
Homes (*n* 92)		16	17·4	48	52·2	28	30·4	4·63	2	0·09
Sports facilities (*n* 11)		0	0·00	7	63·6	4	36·4	–		–
Transportation centres (*n* 65)		13	20·0	37	56·9	15	23·1	7·37	2	0·02*
Food inns (*n* 32)		8	25·0	13	40·6	11	34·4	1·32	2	0·51
Schools (*n* 24)		10	41·7	7	29·2	7	29·2	1·95	2	0·37
Churches (*n* 17)		5	29·4	10	58·8	2	11·8	1·9	2	0·38
Worksites (*n* 43)		7	16·3	18	41·9	18	41·9	0·29	2	0·86
Parks (*n* 13)		6	46·1	5	38·5	2	15·4	1·63	2	0·44
Malls (*n* 11)		0	0·00	11	100	0	0·00	–		–
Restaurants (*n* 12)		0	0·00	6	50·0	6	50·0	–		–
Cereal										
Homes (*n* 117)		26	22·2	52	44·4	39	33·3	0·85	2	0·65
Sports facilities (*n* 14)		0	0·00	5	35·7	9	64·3	–		–
Transportation centres (*n* 73)		20	27·4	30	41·1	23	31·5	2·39	2	0·30
Food inns (*n* 33)		10	30·3	6	18·2	17	51·5	16·4	2	<0·001†,‡
Schools (*n* 88)		14	56·0	2	8·00	9	36·0	7·43	2	0·02‡
Churches (*n* 17)		5	29·4	9	52·9	3	17·6	0·45	2	0·79
Worksites (*n* 44)		7	15·9	10	22·7	27	61·4	12·3	2	0·002†,‡
Parks (*n* 18)		7	38·9	6	33·3	5	27·8	0·06	2	0·97
Malls (*n* 10)		0	0·00	9	90·0	1	10·0	–		–
Restaurants (*n* 11)		0	0·00	2	18·2	9	81·8	–		–

SFS, street food stands; -, calculation not performed due to small sample size.

* Higher distribution in middle- than in low-income neighbourhoods.

† Higher distribution in high- than in low-income neighbourhoods.

‡ Higher distribution in high- than in middle-income neighbourhoods.

The distribution of water across points of access and income levels was limited (Table 5). SFS selling water were often found near homes (46·4 %), transportation centres (45·0 %) and schools (66·7 %) in middle-income neighbourhoods, but these differences were not statistically significant (*P* > 0·05). Regular soda had a high distribution across income levels and points of access compared to other types of beverages. SFS selling regular soda were often found near homes (46·9 %) and transportation centres (47·4 %) in middle-income neighbourhoods, near schools (44·4 %) in low-income neighbourhoods and near parks (50·0 %) in the high-income neighbourhood, but these differences were not statistically significant (*P* > 0·05).

**Table 5:** Distribution of street beverages found at SFS (*n* 391) across neighbourhood income levels and points of access in Mexico City

Type of beverage item	Point of access	Neighbourhood income levels						*X*^2^	df	*P*
		Low		Middle		High				
		*n*	%	*n*	%	*n*	%			
Regular soda										
Homes (*n* 96)		16	16·7	45	46·9	35	36·5	5·15	2	0·07
Sport facilities (*n* 12)		0	0·00	6	50·0	6	50·0	2·98	2	0·22
Transportation centres (*n* 57)		12	21·0	27	47·4	18	31·6	3·97	2	0·13
Food inns (*n* 23)		8	34·8	7	30·4	8	34·8	1·75	2	0·41
Schools (*n* 18)		8	44·4	5	27·8	5	27·8	0·81	2	0·66
Churches (*n* 15)		6	40·0	8	53·3	1	6·7	2·81	2	0·24
Worksites (*n* 43)		7	16·3	17	39·5	19	44·2	0·40	2	0·81
Parks (*n* 18)		5	27·8	4	22·2	9	50·0	4·63	2	0·09
Malls (*n* 12)		0	0·00	11	91·7	1	8·33	0·14	2	0·70
Restaurants (*n* 18)		0	0·00	5	27·8	13	72·2	1·87	2	0·17
Diet soda										
Homes (*n* 6)		2	33·3	2	33·3	2	33·3	0·34	2	0·84
Sports facilities (*n* 0)		0	0·00	0	0·00	0	0·00	–	–	–
Transportation centres (*n* 5)		1	20·0	1	20·0	3	60·0	3·34	2	0·18
Food inns (*n* 3)		1	33·3	2	66·7	0	0·00	1·27	2	0·53
Schools (*n* 1)		1	100	0	0·00	0	0·00	–		–
Churches (*n* 1)		0	0·00	1	100	0	0·00	–		–
Worksites (*n* 6)		1	16·7	1	16·7	4	66·7	1·89	2	0·38
Parks (*n* 3)		0	0·00	0	0·00	3	100	–		–
Malls (*n* 1)		0	0·00	0	0·00	1	100	–		–
Restaurants (*n* 6)		0	0·00	2	33·3	4	66·7	0·15	2	0·69
Water										
Homes (*n* 28)		3	10·7	13	46·4	12	42·9	3·91	2	0·14
Sports facilities (*n* 3)		0	0·00	2	66·7	1	33·3	0·75	2	0·68
Transportation centres (*n* 20)		3	15·0	9	45·0	8	40·0	3·77	2	0·15
Food inns (*n* 7)		0	0·00	5	71·4	2	28·6	3·75	2	0·15
Schools (*n* 3)		0	0·00	2	66·7	1	33·3	4·01	2	0·13
Churches (*n* 4)		0	0·00	4	100	0	0·00	–		–
Worksites (*n* 12)		0	0·00	6	50·0	6	50·0	3·08	2	0·21
Parks (*n* 5)		0	0·00	1	20·0	4	80·0	6·75	2	0·03
Malls (*n* 4)		0	0·00	3	75·0	1	25·0	0·87	2	0·34
Restaurants (*n* 13)		0	0·00	5	38·5	8	61·5	0·03	2	0·84
SSB										
Homes (*n* 84)		20	23·8	33	39·3	31	36·9	1·95	2	0·37
Sports facilities (*n* 14)		1	7·14	6	42·9	7	50·0	1·63	2	0·44
Transportation centres (*n* 54)		13	24·1	23	42·6	18	33·3	3·05	2	0·21
Food inns (*n* 27)		7	25·9	8	29·6	12	44·4	5·45	2	0·06
Schools (*n* 18)		10	55·6	2	11·1	6	33·3	3·21	2	0·20
Churches (*n* 9)		4	44·4	4	44·4	1	11·1	1·20	2	0·54
Worksites (*n* 34)		4	11·8	12	35·3	18	52·9	3·15	2	0·20
Parks (*n* 15)		4	26·7	3	20·0	8	53·3	4·98	2	0·08
Malls (*n* 9)		0	0·00	7	77·8	2	22·2	1·50	2	0·22
Restaurants (*n* 20)		0	0·00	5	25·0	15	75·0	3·25	2	0·07

SSB, sugar-sweetened beverages; SFS, street food stands; -, calculation not performed due to small sample size.

## Discussion

The purpose of this study was to document the availability, variety and distribution of ready-to-eat street food and beverage items in Mexico City and to explore differences in these variables across neighbourhood income levels. Although descriptive patterns in availability, variety and distribution existed by neighbourhood income, very few differed statistically. The availability and variety of unhealthy processed snacks were higher in low-income neighbourhoods with an average of 10·80 different items per stand compared to 4·91 items per stand in middle- and 9·05 items per stand in high-income neighbourhoods (*P* < 0·05). Processed snacks were often found near worksites in high-income neighbourhoods, near transportation centres in middle-income and near schools in low-income neighbourhoods, but there were no statistically significant differences in their distribution (*P* > 0·05). In comparison, although there were no differences in the availability and variety of meat, dairy and cereal items, their distribution by neighbourhood income level was statistically significant. Meat, dairy and cereal items were most often found near food inns, worksites and transportation centres (*P* < 0·05). No statistically significant differences were seen for the other foods and beverages; however, several patterns were observed. Fruit/vegetable availability was high in middle-income neighbourhoods, but their variety was high in high-income neighbourhoods with an average of 5·25 different fruit/vegetable items per stand compared to 4·61 in low- and 5·01 in middle-income neighbourhoods. Availability and variety of water and diet soda were also high in high-income neighbourhoods. For example, the average variety of bottled water per stand was 0·33 in high-income compared to 0·18 in low-income and 0·24 in middle-income neighbourhoods. While diet soda availability and variety were limited across the three income levels, the highest average was seen in high-income (0·08) and the lowest in the low-income neighbourhoods (0·04). Regular soda availability was high in middle-income neighbourhoods, but its variety was high in high-income neighbourhoods with an average of 1·71 types of soda in high-income neighbourhoods compared to 1·29 in middle- and 1·07 in low-income. These findings are partially in line with our initial hypothesis.

We hypothesised that SFS in low-income neighbourhoods would have high availability of unhealthy items such as processed snacks and regular sodas, and we found that this was indeed the case for processed snacks. We also predicted that the availability of healthy items such as fruits/vegetables and water would be high among SFS in high-income neighbourhoods. We found that was indeed the case for water. This study is the first to document SFS food and beverage availability across neighbourhood income levels in a Mexican city using a validated assessment tool. Future research is needed to confirm this study’s results.

The high availability of unhealthy food items such as processed snacks in SFS in low-income neighbourhoods presents a concern for public health practitioners. The consumption of processed snacks (which often have a high content of fat, salt and sugar) is associated with negative health outcomes such as obesity, diabetes and some types of cancer^(54–57)^. In addition, low-income communities are vulnerable populations that may not have access to healthy food items via other venues, such as supermarkets. However, we found it encouraging that when the availability of all food categories was considered within neighbourhood income level rather than across, fruits/vegetables had the highest availability in all three income levels compared to any other food or beverage items, including processed snacks. This finding regarding the availability of healthy food items is in line with previous SFS studies suggesting that street foods can be an important source of nutrients for vulnerable populations, such as residents of low-income communities^(24,27,58–61)^. While our findings suggest that SFS can indeed be a source of healthy food items, such as fruits/vegetables, we do not know which customers the vendors were aiming for with these items or who ultimately purchased and consumed these items. Furthermore, the preparation and cooking methods of street foods should be examined to understand the full nutritional value of street foods and how different preparation methods might improve the healthiness of the food.

Surprisingly, our findings suggest that low-income neighbourhoods are less exposed than middle- and high-income neighbourhoods to unhealthy beverages, such as regular sodas. The low availability of regular sodas in low-income neighbourhoods could be associated with efforts by the Mexican government to curb the consumption of these beverages. In 2014, the Mexican federal government imposed an excise tax on SSB. Following these efforts, the most significant decreases in purchases of regular sodas have been observed in low-income communities^(62)^. Possibly, the higher cost of SSB such as regular soda has reduced the demand for them, and this has led to lower availability of regular soda at SFS in low-income neighbourhoods. More research is needed to confirm these findings.

In terms of differences in food and beverage varieties, our findings showed that the variety (i.e. different types) of processed snacks was higher than that of other food items (*P* < 0·05): it ranged from 0 to 94 items in low-, 0 to 68 items in middle- and 0 to 117 items in high-income neighbourhoods. In comparison, fruit/vegetable items ranged from 0 to 18 in low, 0 to 21 in in middle- and 0 to 26 items in high-income neighbourhoods (*P* > 0·05). This is concerning, as processed snacks have been associated with negative health outcomes^(55,56,63,64)^. Furthermore, some research has suggested that food intake increases as food variety increases^(65,66)^. Thus, a high variety of unhealthy foods may lead to high consumption of those foods. Many of the snacks observed by the RA were small items such as bubble gum and pieces of hard candy, which would not contribute substantial calories to an individual’s diet. However, from a public health perspective, the variety of processed snacks should be reduced and that of healthy foods expanded, particularly since the consumption of healthy foods such as fruits/vegetables can be protective against negative health outcomes^(11,13,63,67)^. Further studies are needed to assess the relationships between food variety, food consumption and individual eating behaviours to determine whether a higher variety of healthy food items at SFS leads to more frequent purchases and higher consumption of those items.

This study’s findings regarding the distribution of ready-to-eat street foods near specific points of access are in line with those from an ethnographic study of SFS in Mexico^(20)^. Our study, however, is the first to report the distribution of ready-to-eat street foods and beverages across neighbourhood income levels. The observed differences in distribution across neighbourhoods depended on the types of food and beverage items. However, we did see similar patterns in the distribution of fruits/vegetables, processed snacks and regular sodas: these foods were frequently found near schools in low-income neighbourhoods; near homes and transportation centres in middle-income neighbourhoods; and near worksites in high-income neighbourhoods, but differences in distribution were not statistically significant. The distribution of unhealthy and healthy foods in the same location is, again, concerning. This is because when both healthy and unhealthy foods were available at the same location, people were more likely to consume unhealthy foods^(68)^. Future studies on ready-to-eat street food and beverage consumption could help guide nutrition interventions and shape the distribution of foods in Mexican communities. It would be important to identify and remove barriers that may be limiting the availability of or access to healthy foods and to implement strategies to discourage unhealthy food availability and consumption. Recently, two Mexican states banned the sale of processed snacks, sodas and other unhealthy foods to anyone under the age of 18, akin to laws banning the sale of alcohol to minors^(69,70)^. It remains to be seen whether this strategy will help to reduce the distribution of unhealthy foods. Alternatively, the versatility and informal nature of street food vending could mean that SFS continue to provide minors with access to unhealthy foods even when regulated businesses (e.g. supermarkets, convenience stores) can no longer sell these products to minors.

### Strengths and limitations

This study has several notable strengths. It is the first study to employ a randomised approach and a validated assessment tool to document the availability and variety of ready-to-eat street foods and beverages across neighbourhood income levels and points of access in a Mexican city. Given the informal nature of street food vending and the unavailability of a SFS business directory, other studies have relied on convenience samples. The methods in this study made it possible to assess a representative sample of SFS and to objectively document the types of foods and beverages being sold there. Previous SFS studies have relied on indirect or intermediate approaches such as interviews and dietary intake recall^(36,37)^, allowing researchers to draw inferences about food and beverage availability, variety and distribution. However, direct observations are preferable, as they can reduce the discrepancies from recall errors or biased responses and thus, would be expected to produce more accurate descriptions of ready-to-eat food and beverage availability. An additional strength of this study is its assessment of different points of access to document the distribution of ready-to-eat street foods and beverages. An advantage of including multiple points of access in the assessments is that it sheds light on which populations the street food vendors may have been targeting. Other studies have described the distribution of street foods and beverages, but those studies have not compared distribution across income levels, or they have focused on only one point of access^(20,34,38,39)^. For example, a couple studies documented the distribution of SFS near schools, and the results of these studies can only be generalised to the immediate vicinity of schools^(34,38,39)^. In contrast, the results from our study can be generalised to a broader population.

At the same time, this study’s limitations must be considered when interpreting its findings. This was an exploratory study; therefore, the power and direction of the relationships between SFS availability, food and beverage availability and variety, and neighbourhood income levels were not established. This study was also cross-sectional. As such, it captured only a snapshot of ready-to-eat food and beverage availability, variety and distribution across time. Food and beverage availability and variety may fluctuate throughout the year. For example, this study conducted SFS assessments from May to August 2018, which would have been the summer break for many school-aged children. Consequently, the distribution of ready-to-eat street food and beverages near schools may have been different during this time compared to when school was in session. A further limitation is that there were some highly mobile street food vendors that we could not include in this study. For example, some vendors were selling food in the middle of the road, right through traffic and we were unable to assess these stands for safety reasons. This may have led to underreporting some types of food/beverage items.

## Conclusion

Documenting the availability, variety and distribution of ready-to-eat foods and beverages sold at SFS can give stakeholders such as health practitioners, policymakers and urban planners useful information to develop strategies for creating healthy food environments. The findings from this study suggest that SFS can be a source of both healthy and unhealthy foods and beverages. Future studies should explore whether SFS, with their versatility and mobility, could focus on needy areas and deliver healthy food items to populations in need of them.
